# Mesenchymal stem cell therapy for acute respiratory distress syndrome: from basic to clinics

**DOI:** 10.1007/s13238-020-00738-2

**Published:** 2020-06-09

**Authors:** Hua Qin, Andong Zhao

**Affiliations:** 1 Research Center for Tissue Repair and Regeneration Affiliated to the Medical Innovation Research Department, PLA General Hospital and PLA Medical College, Beijing, 100853 China; 2 Tianjin Medical University, Tianjin, 300070 China

**Keywords:** mesenchymal stem cells, cell therapy, acute respiratory distress syndrome, SARS-CoV-2, COVID-19, pneumonia

## Abstract

The 2019 novel coronavirus disease (COVID-19), caused by the severe acute respiratory syndrome coronavirus 2 (SARS-CoV-2), has occurred in China and around the world. SARS-CoV-2-infected patients with severe pneumonia rapidly develop acute respiratory distress syndrome (ARDS) and die of multiple organ failure. Despite advances in supportive care approaches, ARDS is still associated with high mortality and morbidity. Mesenchymal stem cell (MSC)-based therapy may be an potential alternative strategy for treating ARDS by targeting the various pathophysiological events of ARDS. By releasing a variety of paracrine factors and extracellular vesicles, MSC can exert anti-inflammatory, anti-apoptotic, anti-microbial, and pro-angiogenic effects, promote bacterial and alveolar fluid clearance, disrupt the pulmonary endothelial and epithelial cell damage, eventually avoiding the lung and distal organ injuries to rescue patients with ARDS. An increasing number of experimental animal studies and early clinical studies verify the safety and efficacy of MSC therapy in ARDS. Since low cell engraftment and survival in lung limit MSC therapeutic potentials, several strategies have been developed to enhance their engraftment in the lung and their intrinsic, therapeutic properties. Here, we provide a comprehensive review of the mechanisms and optimization of MSC therapy in ARDS and highlighted the potentials and possible barriers of MSC therapy for COVID-19 patients with ARDS.

## The epidemiology of novel coronavirus disease 2019 (COVID-19) and the management of ARDS

Since December 2019, an increasing number of patients have been infected by severe acute respiratory syndrome coronavirus 2 (SARS-CoV-2), which causes pneumonia in China (Chan et al., 2020; Chen et al., 2020a; Chinazzi et al., 2020; Ghinai et al., 2020; Guan et al., 2020; Kucharski et al., 2020; Le et al., 2020). The SARS-CoV-2 pneumonia is defined as a novel coronavirus disease 2019 (COVID-19) by World Health Organization (WHO). Human-to-human transmission has been occurring, and infections have been rapidly spreading in China and around the world (Chen et al., 2020a; Chinazzi et al., 2020; Kucharski et al., 2020). Some patients with severe pneumonia rapidly develop acute respiratory distress syndrome (ARDS) and require intensive care unit (ICU) admission (Guan et al., 2020; Holshue et al., 2020; Sun et al., 2020; Xu et al., 2020a). At this stage, patients worsen in a short period and die of multiple organ failures, such as severe respiratory failure, heart failure, and renal failure. As SARS-CoV-2 is an emerging virus infection, the pathological changes happening in severe patients need to be fully understood, and no effective treatment has been developed to prevent worsening of these severe patients and to treat ARDS due to pneumonia. Current management for SARS-CoV-2-infected patients with severe pneumonia and ARDS remains supportive, including anti-infection, intubated ventilator-assisted breathing therapy, and extracorporeal membrane oxygenation (ECMO) (Chen et al., 2020a; Guan et al., 2020; Kandel et al., 2020; Le et al., 2020; Xu et al., 2020a). Since ARDS is associated with high mortality and morbidity, it is urgent to develop new effective approaches to the control of ARDS.

ARDS is a devastating disorder characterized by acute and refractory hypoxia noncardiogenic pulmonary edema, diffuse alveolar-capillary membrane damage, and reduced compliance (Ranieri et al., 2012). Despite decades of research, there is still no effective pharmacotherapy for ARDS. Although some supportive care approaches are established, ARDS remains devastating and life-threatening (Bellani et al., 2016; Papazian et al., 2019).

A growing body of evidence has shown that cell-based therapy holds therapeutic effects for ARDS. Most studies have focused on the therapeutic effects of mesenchymal stem cells (MSCs), although some studies have also investigated the possible applications of pluripotent stem cells, pulmonary epithelial progenitors, and endothelial progenitor cells (Laffey and Matthay, 2017; Han et al., 2019; Lopes-Pacheco et al., 2019). Mounting studies have examined the efficacy of MSC therapy across a wide array of experimental ARDS models (Table 1). Importantly, some clinical trials have been completed or are in progress to evaluate the safety and efficacy of MSC therapy in patients with ARDS and COVID-19, shown in Table 1 (Zheng et al., 2014; Wilson et al., 2015; Matthay et al., 2019; Bing et al., 2020; Leng et al., 2020).

**Table 1:** Preclinical and clinical studies to evaluate therapeutic efficacy of MSCs in ARDS

MSC sources	Disease	Animal or clinical studies	Key findings	References
**hUC-MSCs**	COVID-19	Clinical	Transplantion of hUC-MSCs was well tolerated and promoted the recovery in a 65-year-old female critically ill COVID-19 patients.	Bing et al. (2020)
**Unknown human**	COVID-19	Clinical	Transplantation of MSCs improve the functional outcomes of seven patients with COVID-19 pneumonia, accompanied by the attenuation of inflammation and recovery of the immune system	Leng et al. (2020)
**hM-MSCs**	ARDS induced by the H7N9 virus	Clinical	MSC Transplantation significantly reduced the mortality of the H7N9-induced ARDS	Chen et al. (2020c)
**hAD-MSCs**	ARDS	Clinical (phase I)	Transplantation of MSCs was safe and well-tolerated in the patients.	Zheng et al. (2014)
**hBM-MSCs**	ARDS	Clinical (phase I)	Transplantation of MSCs was tolerated, without adverse effects and differences in the concentrations of IL-6, IL-8, angiopoietin-2, and advanced glycosylation end-product specific receptor (AGER)	Wilson et al. (2015)
**hBM-MSCs**	ARDS	Clinical (phase II)	Transplantation of MSCs improved the oxygenation index and reduced the level of angiopoietin-2 in the plasma.	Matthay et al. (2019)
**hBM-MSCs**	ARDS	Clinical (Compassionate use)	Both patients showed improvement with the resolution of respiratory, hemodynamic, and multiorgan failure. The beneficial effects were associated with a decrease in the biomarkers related to inflammation.	Simonson et al. (2015)
**rLung-MSC**	ARDS induced by LPS	Animal (rat)	Reduced lung inflammation and pulmonary edema. A decrease in IL-1, IL-1 β, IL-6, and TNF-α levels. Restoration of Treg/Th17 balance.	Wang et al. (2019)
**mBM-MSCs**	ARDS induced by HCL instillation	Animal (mouse)	Attenuation of fibrosis in the lung.	Islam et al. (2019)
**mBM-MSCs**	ARDS induced by LPS	Animal (mouse)	Improved the differentiation of MSCs into alveolar epithelial cells. Restoration of the injured structure and function of alveolar epithelial cells. Reduced lung fibrosis.	Zhang et al. (2019c)
**rBM-MSCs**	ARDS induced by LPS	Animal (rat)	Improved oxygen saturation. Reduced lung inflammation and pulmonary edema. Reduced IL-6 and TNF-α levels.	Mokhber Dezfouli et al. (2018)
**rBM-, rAD-** **rlung-MSCs**	ARDS induced LPS	Animal (rat)	Improved lung function and reduced alveolar collapse. Reduced lung inflammation and lung fibrosis. Reduced TNF-α, IL-1β, KC, and TGF-β levels. Reduced apoptosis in the lung, kidney, and liver.	Silva et al. (2018)
**hUC-MSCs**	ARDS induced by LPS	Animal (mouse)	Mitigation of lung injuries. Changing the expression of ARDS-related genes, such as *Cyp17a1*, *Nr1h4*, *Rps6ka6 Nol3*, and *Prkg2*.	Huang et al. (2018)
**hM-MSCs**	ARDS induced by LPS	Animal (mouse)	Reduced lung inflammation and pulmonary edema. Reduced MPO activity and IL-1β level. Increased IL-10 level.	Xiang et al. (2017)
**mAD-MSCs**	ARDS induced by LPS	Animal (mouse)	Improved survival. Reduced lung inflammation. Reduced TNF-α and IL-6 levels.	Pedrazza et al. (2017)
**hUC-MSCs**	ARDS induced by *E*. *coli*	Animal (mouse)	Reduced lung inflammation. Increased bacterial clearance. Reduced alveolar wall thickening. Reduced IL-1α, IL-1β, IL-6, and TNF-α levels.	Sung et al. (2016)
**hBM-MSCs**	ARDS induced by *E*. *coli*	Animal (mouse)	MSCs transfer their mitochondria to macrophages. Increased phagocytosis activity of macrophages.	Jackson et al. (2016)
**hM-MSCs**	ARDS induced by the cecal ligation and puncture	Animal (mouse)	Improved survival. Enhanced bacterial clearance. Reduced inflammation. Reduced TNF-α, MCP1, IL-6, and IL-10 levels.	Alcayaga-Miranda et al. (2015)
**hBM-MSCs**	ARDS induced by *E*. *coli*	Animal (mouse)	Improved lung recovery. Enhanced bacterial clearance. Increased IL-10 and KGF levels. Reduced IL-16 level.	Devaney et al. (2015)

hM, human menstrual blood-derived; hBM, human bone marrow-derived; hAD, human adipose-derived; mBM, mouse bone marrow-derived; hUC, human umbilical cord-derived; mAD, mouse adipose tissue-derived; rlung, rat lung-derived; rBM, rat bone marrow-derived; rAD, rat adipose tissue-derived; *E*. *coli*, *Escherichia coli*; LPS, lipopolysaccharide; IL, interleukin; TNF, tumor necrosis factor; KGF, keratinocyte growth factor; MCP1, monocyte chemoattractant protein 1; TGF, transforming growth factor

In this review, we discussed the mechanisms of MSCs in treating ARDS and the optimization of MSC therapy and highlighted the perspectives related to the therapeutic applications of MSCs in COVID-19 patients with ARDS.

## Mechanisms of MSC adoptive transfer in treating ARDS

**Figure 1:**
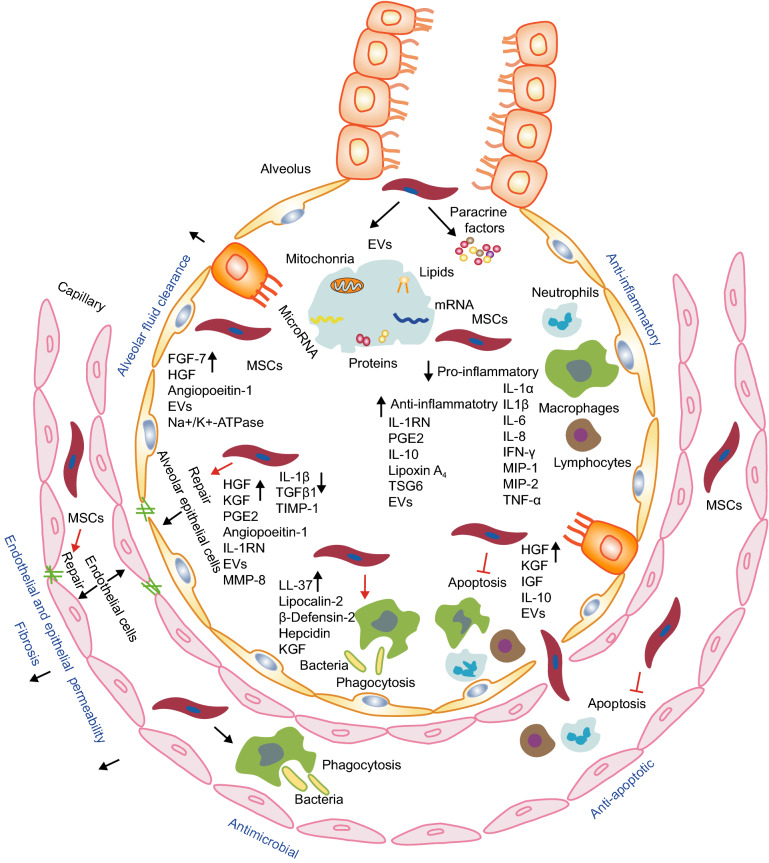
**The mechanisms of MSC therapy in ARDS**. The therapeutic effects of MSCs in ARDS involve multiple mechanisms via their secretion of soluble paracrine protein factors and extracellular vesicles (EVs). MSCs can exert anti-inflammatory, anti-apoptotic, and anti-microbial effects, protect the pulmonary endothelial and alveolar epithelial cells, enhance alveolar fluid clearance, and inhibit lung fibrosis

MSCs are harvested from a variety of sources, including the bone marrow, adipose tissue, placenta, and umbilical cord blood (Le Blanc and Mougiakakos, 2012). MSCs possess specific properties that make them attractive candidates for therapeutic use in kinds of diseases, including inflammatory autoimmune diseases, tissue damage repair, etc. They are non-immunogenic, have low tumorigenicity and short lifespan *in vivo*, possess immunomodulatory and anti-inflammatory effects, and also can detect injured microenvironments and direct their responses for triggering the regeneration process (Stappenbeck and Miyoshi, 2009; Le Blanc and Mougiakakos, 2012). Preclinical and clinical studies have suggested that *in vivo* administration of MSCs exerts anti-inflammatory and anti-apoptotic effects, enhances epithelial and endothelial cell recovery, promotes microbial and alveolar fluid clearance, and reduces lung and distal organ injuries in the treatment of ARDS (Fig. 1).

## Induction of anti-inflammatory response

*In vivo* transfer of MSCs can mitigate inflammation by reducing levels of inflammatory mediators and increasing levels of anti-inflammatory and pro-resolution factors in experimental ARDS models (Fig. 1). The anti-inflammatory effects of MSCs have been mostly attributed to their release of paracrine factors, because of low engraftment of donor-derived MSCs into at the host lung tissue after MSC therapy (Qin et al., 2012; Millar et al., 2019). In support of this, MSC-conditioned media have been suggested to reduce pro-inflammatory mediator levels and inflammatory cell counts in ARDS models (Ionescu et al., 2012; Goolaerts et al., 2014; Chen et al., 2015; Su et al., 2019; Xu et al., 2019). It was recently shown that certain therapeutic effects of MSCs depend on their ability to secrete extracellular vesicles. Extracellular vesicles can transfer messenger RNA (mRNA), microRNA, proteins, lipids, and even organelles such as mitochondria to the target cells and tissues, altering gene expression and modulating the behavior of target cells to attenuate the inflammatory response, consequently mediating and exerting MSC therapeutic effects (Hao et al., 2019; Lee et al., 2019; Abraham and Krasnodembskaya, 2020).

Accumulating evidence demonstrates that patients with severe COVID-19 suffer from the hyperinflammatory syndrome or cytokine storm syndrome, characterized by hypercytokinaemia, such as an increase in the levels of interleukin (IL)-1β, IL-2, Interferon γ-induced protein 10 (IP10), granulocyte colony-stimulating factor (GCSF), IL-7, interferon γ (IFNγ), monocyte chemotactic protein 1 (MCP1), macrophage Inflammatory Protein 1 α (MIP1α), and Tumor necrosis factor α (TNFα) (Chen et al., 2020a; Haberman et al., 2020; Koff and Williams, 2020; Mehta et al., 2020; Schett et al., 2020). A retrospective, multicentre study of 150 COVID-19 patients, showed that the patients that died had higher levels of serum ferritin, C-reactive proteins, and IL-6 as compared to the survivors, indicating that hyperinflammation contributed to death (Schett et al., 2020; Stebbing et al., 2020; Vaninov, 2020). Similarly, a retrospective, multicentre cohort study containing 191 COVID-19 patients also found increased ferritin and IL-6 (Mehta et al., 2020; Vaninov, 2020). Yang et al., conducted a multiplex screen for 48 cytokines in 53 moderate and severe COVID-19 patients and revealed a significant increase in the levels of 14 cytokines in COVID-19 patients as compared with healthy controls. Among them, the increased IP-10, MCP3, hepatocyte growth factor (HGF), monokine induced gamma interferon (MIG), and MIP1α were closely linked to the disease severity (Yang et al., 2020). Moreover, highly proinflammatory monocyte-derived FCN1^+^ macrophages were present in bronchoalveolar lavage fluid of the severe COVID-19 patients but not the mild patients (Liao et al., 2020). A markedly higher number of CD14^+^CD16^+^ inflammatory monocytes were observed in the peripheral blood of severe COVID-19 patients compared to mild cases (Zhou et al., 2020b). These data suggest that anti-inflammatory effects conferred by MSCs might suppress the hyperinflammatory syndrome in severe COVID-19 patients.

## Reduction of cell apoptosis

Apoptosis of both resident and immune cells contribute to ARDS progression, as apoptotic cells recruit inflammatory cells and result in tissue remodeling. *In vivo* administration of MSCs into ARDS models can reduce the apoptotic cell counts in the lung tissues and distal organs in ARDS (Silva et al., 2018). MSCs have also been reported to reduce apoptosis of the alveolar epithelial cells and endothelial cell by secretion of keratinocyte growth factor (KGF), angiopoietin-1, and HGF (Bernard et al., 2018; Chen et al., 2019a; Meng et al., 2019). Alveolar macrophage apoptosis induced by lipopolysaccharide (LPS) was also attenuated after MSC treatment, which may be partially mediated by inhibition of the Wnt/β-catenin pathway (Li et al., 2015). A reduction of tumor necrosis factor-α (TNF-α) that induces cell death may also contribute to the anti-apoptotic effects of MSC therapy (Kim et al., 2011; Güldner et al., 2015).

Lymphocytopenia is common in COVID-19 patients, which mostly impairs the CD4^+^ T cell subsets (Chen et al., 2020b; Goyal et al., 2020; Yang et al., 2020). The apoptosis of lymphocytes induced by SARS-CoV-2 infection leads to lymphocytopenia in critically ill patients with COVID-19 pneumonia (Chen et al., 2020b; Zheng et al., 2020). Mild patients have only mild lymphocytopenia, whereas severe patients have severe lymphocytopenia, suggesting apoptosis of lymphocytes reflects the severity of COVID-19 pneumonia to some extent. Moreover, SARS-CoV-2 infection results in apoptosis of alveolar epithelial cells and endothelial cells (Monteil et al., 2020; Varga et al., 2020; Walls et al., 2020). For this reason, MSC-based therapy may bring about beneficial effects for COVID-19 patients possibly by inhibiting apoptosis of resident cells and immune cells.

## Initiation of the anti-microbial innate response

MSCs therapy has been proven to promote bacterial clearance in the lung in animal models of ARDS. Such effects of MSCs are associated with the secretion of anti-microbial peptides and proteins, including LL-37, lipocalin-2, and β-defensin-2, hepcidin, and KGF (Krasnodembskaya et al., 2010; Gupta et al., 2012; Alcayaga-Miranda et al., 2015; Sung et al., 2016). Alternative mechanisms underlying the bacterial clearance by MSCs include, at least partially, the enhancement of the phagocytic activity of macrophages and monocytes via promoting mitochondrial transfer via tunneling nanotubes in the model of ARDS (Krasnodembskaya et al., 2012; Lee et al., 2013; Jackson et al., 2016). Therefore, these studies together show that MSCs can enhance the innate immune responses against bacterial infection via direct and indirect mechanisms.

Some critically ill patients with COVID-19 had co-infections of bacteria and fungi. A retrospective study of 113 deceased patients showed many of them developed secondary bacterial infections, which were highly associated with death (Chen et al., 2020b). Another study of 339 elderly patients with COVID-19 showed that about 42% of the patients developed bacterial infections, and bacterial infection was increased significantly in dead patients (Wang et al., 2020a). Empirical antibacterial therapy could promote recovery (Chen et al., 2020b). It is speculated that MSC transplantation may mitigate bacterial infections in severe patients with COVID-19.

## Protection of pulmonary endothelial cells and alveolar epithelial cell damage

Breakdown of alveolar-capillary membrane integrity is a hallmark of ARDS, which contributes to edema formation and tissue remodeling. MSC therapy has been shown to preserve or restore the alveolar epithelial and pulmonary endothelial lining by secreting HGF via extracellular vesicles, reducing inflammatory damage, and increasing autophagy (Yang et al., 2015b; Zhou and You, 2016; Hu et al., 2018; Meng et al., 2019). MSC therapy has also been demonstrated to differentiate directly into type II alveolar epithelial cells for the regeneration by activation of wnt/β-catenin signaling (Liu et al., 2013; Cai et al., 2015; Li et al., 2017; Zhang et al., 2019b). In addition to differentiation into alveolar epithelial cells, MSCs can protect the alveolar epithelial cells against the inflammation-induced damage and oxidative injuries by secretion of angiopoietin-1, interleukin 1 (IL-1) receptor antagonist (IL-1RN), prostaglandin E2 (PGE2), HGF, and KGF or scavenging oxidants and radicals (Fang et al., 2010; Goolaerts et al., 2014; Bernard et al., 2018; Yan et al., 2019). Moreover, MSCs can regulate tissue remodeling processes and attenuate the lung fibrosis by increasing metalloproteinase (MMP)-8 and decreasing the levels of tissue inhibitor of metalloproteinase (TIMP)-1, IL-1 β, and transforming growth factor-β1 (TGF-β1) in the animal model of ARDS (Maron-Gutierrez et al., 2013; Silva et al., 2018). Taken together, MSCs can protect pulmonary endothelial and epithelial cell damage and restore their impaired barrier functions.

The SARS-CoV-2 infects the host using the angiotensin-converting enzyme II (ACE2) receptor (Lan et al., 2020; Shang et al., 2020; Wang et al., 2020b; Yan et al., 2020; Yuan et al., 2020; Zhao et al., 2020; Zhou et al., 2020a), which is widely expressed on the alveolar epithelial type II cells and the endothelial cells in the lung and many other organs, including the heart, liver, kidney, and intestines. In patients with COVID-19, post-mortem analysis demonstrated that the SARS-CoV-2 infected the endothelial cells in the lungs, kidneys, intestines, hearts, and livers and caused endotheliitis and endothelial apoptosis (Varga et al., 2020). Moreover, the SARS-CoV-2 binds to endothelial cells in the lungs and subsequently activates bradykinin 1 receptor (B1R) and B2R on endothelial cells, which result in lung angioedema (van de Veerdonk et al., 2020). *In vitro*, SARS-CoV-2 can infect the human-engineered blood vessel organoids (Monteil et al., 2020). Alveolar epithelial type II cells produce surfactants that can reduce surface tension, which is essential for the gas exchange function. SARS-CoV-2 viruses infect alveolar epithelial type II cells and result in severe injuries to them in COVID-19 patients (Walls et al., 2020; Zhao et al., 2020; Zhou et al., 2020a). The observation of severe injuries to endothelial cells and alveolar epithelial type II cells by SARS-CoV-2 infection may provide a rationale for MSC transplantation therapy to protect the endothelial cells and alveolar epithelial cells in COVID-19 patients.

## Improvement of alveolar fluid clearance

Removal of excessive alveolar and interstitial fluid facilitates lung function recovery in ARDS, as fluid impairs surfactant concentration and gas exchange. MSCs can improve alveolar fluid clearance by the secretion of paracrine factors and regulating the function of membrane channels and transporters. *In vivo* administration of MSCs has been shown to increase the alveolar fluid clearance in ARDS models and in *ex vivo* perfused human lung that is injured by endotoxin or Escherichia coli (Lee et al., 2013; Hao et al., 2015; Park et al., 2019; Zhang et al., 2019b). These effects may be associated with the secretion of fibroblast growth factor-7 (FGF-7) and extracellular microvesicles (Park et al., 2019). In a lung injury model induced by influenza infection, treatment with MSCs promotes the alveolar fluid clearance by secreting angiopoietin-1 and KGF and suppressing the downregulation of Na^+^/K^+^-ATPase (Chan et al., 2016). In the bronchoalveolar lavage fluid collected from COVID-19 patients, a large number of SARS-CoV-2 viruses are detected (Lu et al., 2020; Zhou et al., 2020a), and highly inflammatory monocyte-derived FCN1^+^ macrophages are present and responsible for producing enormous cytokines (Liao et al., 2020), indicating inflammatory microenvironment in the alveolar fluid. Accordingly, the promotion of alveolar fluid clearance by MSC transplantation may possibly contribute to the recovery of lung function in severe patients with COVID-19.

## Attenuation of lung and distal organ injuries

Multiple organ failure can occur as ARDS aggravates, which ultimately results in increased morbidity and mortality. MSC administration can reduce the histopathological impairment of lung tissues and promote the functional recovery in the ARDS models (Mokhber Dezfouli et al., 2018; Silva et al., 2018; Zhang et al., 2019b). Administration of MSCs can also improve the repair and function recovery in other distal organs, including heart (Lee et al., 2009; Golpanian et al., 2016), liver (Lee et al., 2018; Silva et al., 2018), kidney (Perico et al., 2017; Silva et al., 2018), and gut (Garcia-Olmo and Schwartz, 2015; Molendijk et al., 2015). Therefore, MSC administration may not only improve the lung function recovery but also delay or suppress the progression of ARDS into multiple organ injuries. Since ACE2 is expressed in other many organs in addition to lungs, SARS-CoV-2 infection leads to injuries to multiple organs, including heart, kidney, liver, and nervous systems (Li et al., 2020; Ronco and Reis, 2020; Wang et al., 2020a; Xu et al., 2020b; Zhang et al., 2020). Given the confirmed beneficial effects for multiple organs after MSC transplantation, MSCs may be used to prevent and mitigate multiple organ failure in COVID-19 patients.

## Strategies to optimize MSC therapy in ARDS

**Figure 2:**
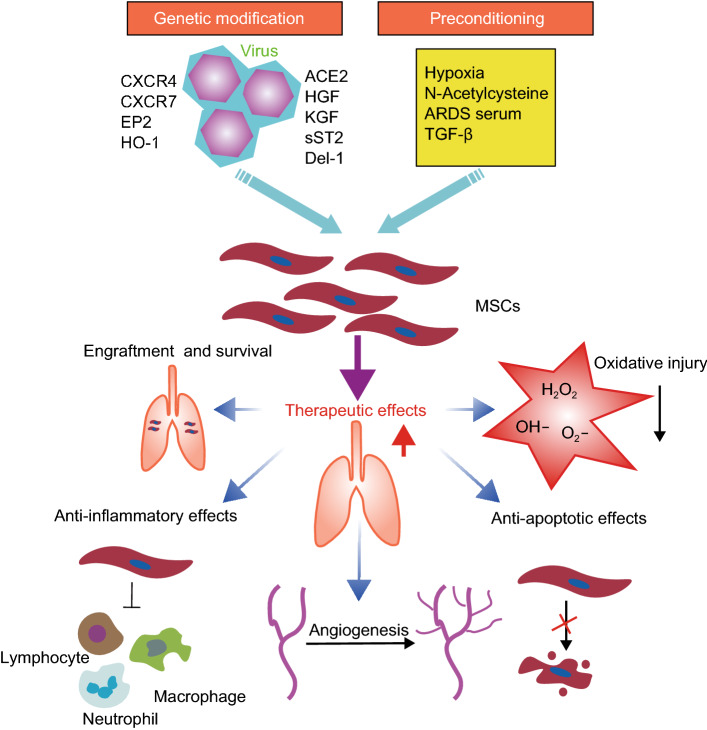
**The strategies to optimize MSC therapy in ARDS**. MSCs can be genetically modified to overexpress beneficial genes or pre-treated with a series of preconditioning strategies, which can promote their therapeutic effects. The improvement of therapeutic effects may depend on an increase in the engraftment and survival of MSCs in the lung, a decrease in the oxidative injury, and enhanced effects of anti-inflammation, anti-apoptosis, and angiogenesis

Low engraftment and poor survival of transplanted MSCs are obstacles for clinical translation of this therapy. Several strategies have been developed to improve MSC engraftment and survival in the lung in the experimental models of ARDS (Fig. 2).

The strategies using genetic modification to overexpress beneficial genes on MSCs have enhanced the migration, survival, and therapeutic potential of MSCs transfer in the models of ARDS (Han et al., 2019). Stromal-derived factor-1 (SDF-1) and the chemokine receptor 4 (CXCR4) signaling axis directs the migration of MSCs. However, low expression of CXCR4 on MSCs impairs their homing to the damaged tissues. Overexpression of *CXCR4* via viral vector has increased the homing and engraftment of MSCs into the injured lung of LPS-induced lung injury (ALI) or radiation-induced ALI models (Yang et al., 2015a; Zhang et al., 2019a). Similarly, overexpression of another SDF-1 receptor *Cxcr7* in MSCs also enhances the therapeutic effects on LPS-ALI model (Shao et al., 2019). PGE2, released in the inflammatory site, can promote the migration of MSCs via activation of the E-prostanoid 2 (EP2) receptor (Goolaerts et al., 2014; Zhu et al., 2017; Xu et al., 2019). MSCs overexpressing EP2 increase their migration to the sites of lung injury in the mouse model of LPS-ALI (Han et al., 2019). In addition to improving the migration of MSCs, genetic modification enhances the intrinsic capacity of MSCs to reduce inflammation and apoptosis. Heme oxygenase-1 (HO-1) is a stress-response protein that has anti-inflammatory, anti-apoptotic, and anti-oxidative effects. Compared to the unmodified MSCs, MSCs transfected with *HO-1* gene show higher potential to ameliorate cytokine levels in serum and the neutrophil counts and total protein concentration in the bronchoalveolar lavage fluid, and improve the histological structure of the injured lung in the LPS-induced ALI rat model (Chen et al., 2018; Chen et al., 2019b). Angiotensin-converting enzyme 2 (ACE2) can protect against lung injury by degrading the profibrotic peptide angiotensin II. Overexpression of the *ACE2* gene in bone marrow-derived MSCs has been reported to increase the potential of MSCs to migrate to lung tissue, mitigate the inflammation, and endothelial injury in the LPS-induced ALI mouse model (He et al., 2015; Xu et al., 2018). Growth factors, such as HGF (Hu et al., 2016; Chen et al., 2017a; Meng et al., 2019), KGF (Chen et al., 2013), and angiopoietin-1 (Mei et al., 2007; Xu et al., 2008; Shao et al., 2018), have shown pro-angiogenic, anti-inflammatory, anti-oxidative, and pro-proliferation effects. MSCs modified with these growth factor genes have significantly improved their therapeutic effects on lung injury. Overexpression of anti-inflammatory and anti-oxidative molecules, including soluble IL-1 receptor-like-1 (*sST2*) (Martínez-González et al., 2013), developmental endothelial locus-1 (*Del-1*) (Zhao et al., 2014), and manganese superoxide dismutase (Chen et al., 2017b), in MSCs also have shown an enhanced ability to treat lung injury.

Nevertheless, viral transfection is associated with the risk of triggering oncogenes and tumorigenesis. Moreover, the establishment of genetically modified MSCs is time-consuming, and it may be hard to administrate genetically modified MSCss immediately following the onset of ARDS. An alternative approach, such as preparation and modification of allogenic MSCs to set up a cell bank for the application needs to be established.

A series of preconditioning strategies have been developed to enhance the therapeutic capacity of MSCs in animal ARDS models. Hypoxic culture of MSCs can stimulate the secretion of bioactive molecules, including vascular endothelial growth factor (VEGF), angiopoietin 1, HGF, insulin-like growth factor 1 (IGF-1), and bal-2, which are associated with pro-angiogenic, anti-apoptotic, and anti-oxidative effects (Chacko et al., 2010; Zhang et al., 2014). Treatment with hypoxia-conditioned MSCs also extends the survival of engrafted cells, improves pulmonary respiratory functions, and reduced inflammatory and pro-fibrotic factor production (Lan et al., 2015). Pre-treatment with N-Acetylcysteine, a precursor of glutathione with anti-oxidative effect, has been demonstrated to strengthen the therapeutic potential of MSCs in ALI mouse models by increasing MSC engraftment and survival rate in the lung tissue (Wang et al., 2013). Pre-activation with serum obtained from ARDS patients enhances the anti-inflammatory capacity of MSCs, evidenced by an increase in the secretion of IL-10 and IL-1-RN (Bustos et al., 2013). Moreover, the serum-preactivated MSCs are more effective in reducing lung injury scores, inflammatory cell counts, and pulmonary edema after transplantation into a mouse model of ARDS (Bustos et al., 2013). Moreover, pretreatment with low levels of TGF-β1 has been shown to increase the MSC survival in the lung following transplantation in a rat model of LPS-induced ALI (Li et al., 2016). These preconditioning strategies might be promising for increasing MSC potency in the management of ARDS.

## Clinical trials of MSC therapy in patients with ARDS

To date, 13 clinical trials assessing the safety and efficacy of MSC therapy in ARDS patients have been registered in the US National Institutes of Health (https://clinicaltrials.gov). Three clinical trials have been completed. The first study to evaluate the safety of MSCs in treating ARDS patients was conducted in China (NCT01902082). Zheng et al., conducted a phase I, single-center, randomized, double-blind, placebo-controlled clinical trial, in which ARDS patients received an intravenous injection of allogenic adipose-derived MSCs (1 × 10^6^ cells/kg) (Zheng et al., 2014). The *in vivo* administered MSCs seemed to be safe and well-tolerated in the patients. The two groups showed no significant difference in the length of hospital day, ventilator-free days, and ICU-free days within 4 weeks after the treatment (Zheng et al., 2014). Wilson et al., conducted another multicenter, open-label, dose-escalation, phase I clinical trials in the USA (NCT01775774) (Wilson et al., 2015). Patients with moderate-severe ARDS received a single intravenous injection of allogeneic bone marrow-derived MSCs at 1, 5, 10 × 10^6^ cells/kg. All dose levels were tolerated, without administration-related adverse effects. No significant differences were observed in the concentrations of related biomarkers such as IL-6, IL-8, angiopoietin-2, and advanced glycosylation end-product specific receptor (AGER) (Wilson et al., 2015). The same group continued to conduct phase IIa clinical trials, in which patients received a high dose level of allogeneic bone marrow MSCs (10 × 10^6^ cells/kg) (NCT02097641) (Matthay et al., 2019). No MSC-related hemodynamic and respiratory adverse effects were observed in all patients. Patients receiving MSC treatment showed an improvement in the oxygenation index. A reduced level of angiopoietin-2 in the plasma was found, indicating that MSC administration attenuated endothelial injury. In a Swedish case report, two patients with severe, refractory ARDS received an intravenous infusion of allogeneic bone marrow-derived MSCs (2 × 10^6^ cells/kg) at a compassionate use (Simonson et al., 2015). Both patients showed improvement with the resolution of respiratory, hemodynamic, and multiorgan failure. The beneficial effects were associated with a decrease in the biomarkers related to inflammation (Simonson et al., 2015). In addition, transplantation of menstrual blood-derived MSCs could reduce the mortality in patients with H7N9 virus-induced ARDS without adverse effects after the five-year follow-up period in China (Chen et al., 2020c). Because H7N9 and COVID-19 share similar complications, MSC transplantation may be useful for treating COVID-19.

In China, about 10 clinical trials have been registered in the Chinese Clinical Trial Registry (http://www.chictr.org.cn/) to investigate the safety and efficacy of transplantation therapy of bone marrow or umbilical cord mesenchymal stem cells for COVID-19 patients with severe pneumonia or ARDS. Liang and colleagues reported that transplantation of human umbilical cord-derived MSCs could modulate the immune response and promote the functional recovery in a 65-year-old female patient with critically ill COVID-19 and severe complications such as respiratory failure and multiple organ failure (Bing et al., 2020). The patient received three doses of 5 × 10^7^ million allogeneic umbilical cord-MSCs every three days. Following the second dose, the vital signs were improved, and she did not require the ventilator. Two days after the third dose, she was transferred out of the ICU. Recently, one published study involved 7 COVID-19 patients (1 critically serious ill, 4 serious ill, and 2 commons) in the MSC-treated group and 3 patients in the control group, with a 14-day follow-up (Leng et al., 2020). In the MSC-treated group, patients received one dose of MSC injection at 1 × 10^6^ cells per kilogram of weight. No serious adverse side effects were observed in the MSC-treated groups. All the 7 patients in the MSC-treated group recovered. A few days after MSC transplantation, oxygen saturation and biomarkers for inflammation were decreased, and peripheral lymphocytes were increased. Moreover, transplanted MSCs were negative for ACE2, indicating MSCs are free from SARS-CoV-2 infection. This study suggests that MSCs can suppress hyperinflammation and improve the immune system. Some other study groups have also reported beneficial effects of MSC therapy for COVID-19 before the publication of their data. Accordingly, Atluri and colleagues consider MSCs as a potential alternative therapy for treating critically ill COVID-19 patients (Atluri et al., 2020).

## Challenges for clinical use of MSC therapy in ARDS

Despite remarkable advances in the control of ARDS with MSC therapy, further research is needed to elucidate several issues, including the optimal MSC source and dose, the time window of MSC administration, administration routes, and frequency (single vs. multiple-dose regimen). Although bone marrow is the most common source for isolating MSCs, the harvesting procedure is invasive, and the cell numbers are limited. Moreover, ARDS affects the immunomodulatory effects of bone marrow MSCs and impairs their potential use for autologous transplantation (Antebi et al., 2018). Although several studies have evaluated the therapeutic effects of MSCs from other sources in ARDS (Zheng et al., 2014; Ren et al., 2018; Silva et al., 2018), it remains unclear about which one may provide superior therapeutic effects. Cell doses are critical for the clinical use of MSC therapy. In experimental models, the number of MSCs administered at a single dose range from 5 × 10^4^ to 3.6 × 10^7^ cells (McIntyre et al., 2016). In the clinical setting, this range in a 25-g mouse would correspond to 2 × 10^6^ to 1.44 × 10^9^ cells/kg in humans. Such quantities are faced with technical and operational challenges. Of note, some safety concerns may be associated with the use of high doses of MSCs. To date, 1 × 10^7^ cells/kg is the highest dose ever used in clinical studies. Accordingly, the optimal number of MSCs administered should be clearly defined for treating ARDS to find a balance between therapeutic effects and undesired safety events. Although a single dose of MSCs has been shown to provide therapeutic benefits, more than one dose may be required to induce a more efficient tissue repair or even to maintain benefits.

The therapeutic window and index for MSC therapies should be further characterized in experimental studies of ARDS. MSC administration has been performed by either local or systemic routes in different experimental models. Local administration via intratracheal infusion delivers cells directly to the site of injury, whereas systemic administration via intravenous infusion allows wide distribution throughout the body. MSCs administered intravenously would encounter the first-class pulmonary effect (Fischer et al., 2009), which results in significant retention of cells in the lung, thereby providing advantages for lung tissue repair. Therefore, ongoing clinical trials and most experimental studies have used the intravenous route for MSC administration. ECMO has been a common therapeutic strategy for patients with severe ARDS. However, MSCs administered intravenously were found to adhere to membrane oxygenator fibers during ECMO in an *ex vivo* model of ARDS, resulting in a significant reduction of flow through the circuit (Millar et al., 2019). Thus, MSC transfer should be performed before ECMO or during a pause in the flow or by intratracheal infusion. Especially for patients with severe ARDS requiring continuous high-flow ECMO, intratracheal administration may be optional for this clinical situation.

## Conclusions and perspectives

Tremendous progress has been made in investigating MSC-based therapy in experimental ARDS models and patients with ARDS. The safety of MSC therapy has also been demonstrated in early-stage clinical studies with a relatively small number of patients.

**Figure 3:**
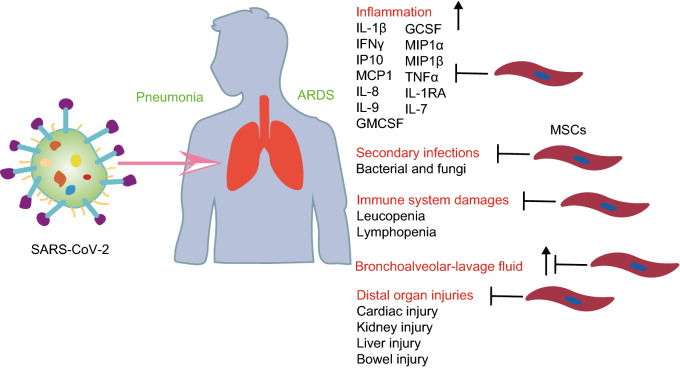
**The potential of MSC-based therapy in COVID-19 patients with severe pneumonia and ARDS by targeting pathophysiological changes**. SARS-CoV-2 infections caused severe pneumonia and ARDS, with significant pathophysiological changes, including inflammation, immune system damages (leukopenia and lymphopenia), secondary infections, and distal organ injuries. However, MSCs have the potential to target these pathophysiological events, acting as a alternative strategy for treating COVID-19 patients with ARDS

SARS-CoV-2 infections caused severe pneumonia and ARDS, with significant pathophysiological changes (Fig. 3). Pulmonary inflammation and extensive lung injury are detected, evidenced by high levels of proinflammatory cytokines in serum (eg., IL-1β, IFNγ, IP10, MCP1, GCSF, MIP1α, MIP1β, and TNFα) (Cao et al., 2020; Chen et al., 2020a; Haberman et al., 2020; Mehta et al., 2020; Schett et al., 2020). Moreover, patients can have secondary infections. In view of the high levels of cytokines induced by SARS-CoV-2 infections, MSC therapy is likely to reduce inflammation-induced lung injury in SARS-CoV-2-infected patients with ARDS. In addition, the blood counts of patients showed leukopenia and lymphopenia, indicating that SARS-CoV-2 consumes immune cells (especially, lymphocytes) and impairs the body’s immune system functions (Cao et al., 2020; Chen et al., 2020a). Administration of MSCs may promote the recovery of the mounts of white blood cells and lymphocytes because MSCs have shown immunomodulatory and anti-apoptotic effects that protect macrophages, neutrophils, and monocytes against apoptosis. Patients with SARS-CoV-2–infected pneumonia may become worse to develop distal organ injuries such as acute cardiac injury, acute kidney injury, liver injury, and bowel injury (Chan et al., 2020; Chen et al., 2020a; Zhu et al., 2020). Given that the systemic administration of MSC has shown protective effects on these distal organs, MSC treatment may decrease the progression of patients with ARDS into multiple organ failure. Apart from the above benefits, MSC can enhance epithelial and endothelial recovery and promote microbial and alveolar fluid clearance by their paracrine secretion, transfer of extracellular vesicles, or cell-cell contact. Therefore, MSC-based therapy may be a potential alternative strategy for treating COVID-19 patients with ARDS.
